# Outbreak of *Clostridium difficile* PCR ribotype 027 - the recent experience of a regional hospital

**DOI:** 10.1186/1471-2334-14-209

**Published:** 2014-04-17

**Authors:** Mónica Oleastro, Marta Coelho, Marília Gião, Salomé Coutinho, Sandra Mota, Andrea Santos, João Rodrigues, Domitília Faria

**Affiliations:** 1 National Reference Laboratory for Gastrointestinal Infections, Department of Infectious Diseases, National Institute of Health Dr. Ricardo Jorge, Av. Padre Cruz, 1649-016 Lisbon, Portugal; 2 Infection Control Team, Centro Hospitalar do Algarve – Unidade Hospitalar de Portimão, Sítio do Poço Seco, 8500-338 Portimão, Portugal; 3 Hospital Pharmacy, Centro Hospitalar do Algarve – Unidade Hospitalar de Portimão, Sítio do Poço Seco, 8500-338 Portimão, Portugal

**Keywords:** *Clostridium difficile*, Outbreak, PCR ribotype 027, Portugal

## Abstract

**Background:**

*Clostridium difficile* infection (CDI) is the leading cause of healthcare-associated diarrhea, and several outbreaks with increased severity and mortality have been reported. In this study we report a *C. difficile* PCR ribotype 027 outbreak in Portugal, aiming to contribute to a better knowledge of the epidemiology of this agent in Europe.

**Methods:**

Outbreak report with retrospective study of medical records and active surveillance data of all inpatients with the diagnosis of CDI, from 1^st^ January to 31^th^ December 2012, in a Portuguese hospital. *C. difficile* isolates were characterized regarding ribotype, toxin genes and moxifloxin resistance. Outbreak control measures were taken, concerning communication, education, reinforcement of infection control measures, optimization of diagnosis and treatment of CDI, and antibiotic stewardship.

**Results:**

Fifty-three inpatients met the case definition of *C. difficile*-associated infection: 55% males, median age was 78.0 years (interquartile range: 71.0-86.0), 75% had co-morbidities, only 15% had a nonfatal condition, 68% had at least one criteria of severe disease at diagnosis, 89% received prior antibiotherapy, 79% of episodes were nosocomial. CDI rate peak was 13.89/10,000 bed days. Crude mortality rate at 6 months was 64.2% while CDI attributable cause was 11.3%. Worse outcome was related to older age (*P* = 0.022), severity criteria at diagnosis (leukocytosis (*P* = 0.008) and renal failure), and presence of fatal underlying condition (*P* = 0.025). PCR ribotype 027 was identified in 16 of 22 studied samples.

**Conclusions:**

This is the first report of a 027-CDI outbreak in Portugal. We emphasize the relevance of the measures taken to control the outbreak and highlight the importance of implementing a close and active surveillance of CDI.

## Background

*Clostridium difficile* is an environmental organism that may be carried asymptomatically in the human intestine, or cause disease in susceptible persons. Those include individuals with disturbed intestinal flora, especially due to recent use of broad-spectrum antibiotics, and/or immunocompromised patients. Also, the risk of *C. difficile* infection (CDI) is higher among elderly patients who had recent contact with healthcare settings [1-4]. Symptoms of CDI range from mild diarrhea to pseudo-membranous colitis, toxic mega colon or even death. CDI is presently the leading cause of healthcare-associated diarrhea in Europe and in North America, representing an enormous clinical and economic burden [5]. It is also an emerging pathogen in the community, and is spreading as well to animals used in food industry [6].

Since 2003, large outbreaks of more severe CDI-associated disease, with higher relapse rates and increased mortality have been reported in North America and Europe, which have been attributed to strains of *C. difficile* belonging to ribotype 027 [7]. These strains were shown to be resistant to fluoroquinolones [3], and displayed genetic mutations in a toxin regulator gene (*tcdC*) causing higher expression of toxins A and B, and produced a binary toxin [8,9]. Nevertheless, the contribution of these factors to the higher virulence of *C. difficile* is yet to be clarified.

Recently, a large hospital-based survey conducted between 2008 and 2009, involving 106 laboratories in 34 European countries, showed that ribotype 027 was not included among the most prevalent European types, with its overall prevalence showing a slight decrease since the last study (5%) [10]. In that survey, the 027 ribotype was not detected among the isolates from Portuguese patients, but only 12 isolates were studied.

This study reports the first outbreak of *C. difficile* due to ribotype 027 strain in a Portuguese hospital, aiming to contribute to a better knowledge of the epidemiology of this agent in Europe, and discusses the bundle of outbreak control measures taken.

## Methods

The present study was considered a retrospective analysis of medical records in the context of an outbreak investigation. Therefore, its execution was communicated to the Ethics Committee of the Centro Hospitalar do Algarve (CHA), but no ethical approval was required.

### Setting

This study was conducted in the Centro Hospitalar do Algarve (CHA), which is a regional secondary hospital in the South of Portugal, with a capacity of 330 beds, including 40 beds (part of the Internal Medicine Department) located separately, 17 km away from the main building. It serves a population of about 170,000 inhabitants and has an Intensive Care unit (12 beds) and Oncology and Haematology Outpatient units (excluding acute leukemia and bone marrow transplant patients). During the year of 2012, the hospital recorded 15 716 inpatient admissions, with a mean length of stay of 7.17 days.

The CHA comprises an Infection Control Program and Infection Control Team (ICT) and written guidelines for the prevention and treatment of CDI since 2008 that are reviewed every 3 years, as well as for the management of outbreaks. The subject “CDI” is discussed every year in the educational activities for the healthcare workers (HCW) and active surveillance of CDI is conducted since 2009, with a mean incidence rate below 2/10,000 patient bed days until the end of the 1^st^ trimester of 2012.

### Data collection and population

We conducted a retrospective study of the medical records and active surveillance data collected by the ICT of all inpatients with the diagnosis of CDI in CHA, during the period from 1^st^ January to 31^th^ December 2012. Demographic, epidemiological and clinical data, co-morbid conditions, antibiotics and proton pump inhibitors (PPI) consumption within the three months prior to diagnosis of CDI, as well as treatment, rate of relapse and 30 day crude and attributable mortality, were registered and analysed in a Microsoft EXCEL™ database.

### Definitions

For case definition, severe disease criteria, and treatment guidelines, the European Society of Clinical Microbiology and Infectious Diseases (ESCMID) treatment guidance for *C. difficile* infection was used [11].

CDI onset classification was defined according to the time of symptom onset and timing of hospitalization in CHA, as summarized elsewhere [12,13].

The Mccabe score was used to evaluate the vital prognosis of the underlying clinical situation, and comprises three categories: nonfatal disease (five year survival not affected by underlying disease), ultimately fatal (not expected to survive more than five years) and rapidly fatal (not expected to survive more than one year) [14].

The incidence rate was calculated as the number of cases per 10,000 patient bed days, according to the studies conducted by the ESCMID [10].

### Outbreak control measures

**Figure 1:**
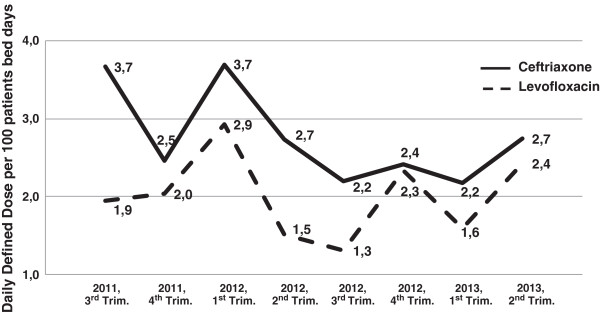
Evolution of the consumption of ceftriaxone and levofloxacin in the hospital from the third trimester of 2011 till the end of 2013.

In April 2012, when the CDI outbreak was declared in the hospital, the ICT Coordinator assumed the leadership of the Outbreak Control Team (OCT), and a bundle of outbreak control measures were taken, concerning five main areas [15,16] – communication, education, reinforcement of infection control measures, optimization of diagnosis and treatment of CDI, and antibiotic stewardship, as follows: i) Communication of the outbreak to local, regional and national public health authorities, with weekly updated reports in the first three months and monthly afterwards, until January 2013; ii) establishment of a network with the Regional Infection Control Group, assuring that the same control measures were adopted by the two main hospitals and all other primary care ambulatory and inpatient institutions, and that all healthcare workers involved received education on the diagnosis, treatment and prevention of CDI; iii) communication via e-mail to all nurse and medical ward leaders, as well as intranet communication at hospital level to inform all healthcare workers of the outbreak, with epidemiological and clinical explanations of the situation; iv) implementation of a bundle of infection control measures according to the institution guidelines, based on several issued international guidelines [12,17-19] - universal precautions including hand hygiene with soap and water and gloves and aprons use, contact isolation precautions including admission to individual room if available or cohorting of confirmed cases (two and three bed rooms), cleaning and disinfecting of wards and equipment every eight hour shift with sodium hypochloride (5,000 ppm concentration) and, after discharge of the patient, peroxide hydrogen vaporization; v) reinforcement of the knowledge of CDI control guidelines, through educational sessions, together with providing educational written material to patients and visitors; vi) implementation of a protocol of procedures in all patients presenting with diarrhea, for an early identification of suspected cases of CDI, including identification of severity criteria and treatment algorithm when confirmed; vii) confirmed toxin positive samples were sent to the National Reference Laboratory of the Portuguese National Institute of Health (NIH) for strain culture typing; viii) antibiotic stewardship - clinicians were reminded to resume their prescriptions of antibiotics and PPIs to those strictly indicated and to adequate the length of antibiotherapy to the clinical situation, and were specifically asked to reduce their prescriptions of quinolones and 3^rd^ generation cephalosporins. The ICT evaluated the compliance to the antibiotic stewardship comparing the consumption of these classes of antibiotics in the hospital before, during and after the outbreak (Figure 1).

### Laboratory diagnosis of *Clostridium difficile* infection

The fecal toxin was determined using the Vidas *C. difficile* Tox A and B assay (bioMérieux, Marcy-l’Etoile, France).

### *Clostridium difficile* culture and strains’ characterization

From 1^st^ July 2012, diarrheal faecal samples of patients hospitalized in different affected wards, with a positive toxin test, were sent to the NIH reference laboratory.

The faecal samples were cultured, after ethanol shock, on the chromogenic medium chromID® *C. difficile* (bioMérieux, Marcy l’Etoile, France), under anaerobic conditions. *C. difficile*-suspected colonies were obtained from all patients, which were identified with the Rapid ID32 A galleries (bioMérieux), and confirmed by detection of *gluD* gene, which encodes the glutamate dehydrogenase specific for *C. difficile*[20].

The isolates were further characterized by capillary gel electrophoresis-based PCR ribotyping, as well as by PCR detection of enterotoxin A and cytotoxin B (*tcdA* and *tdcB* genes, respectively), binary toxin (*cdtA* and *cdtB* genes), and the deletion in the *tcdC* gene [21,22]. The susceptibility to moxifloxacin was determined by E-Test® (bioMérieux).

### Statistical analyses

Non normally distributed variables were compared with the Mann–Whitney test. Proportions were compared with the χ2 test. The *P* values of <0.05 were considered significant.

## Results

### Patient’s characterization

From 1^st^ January to 31^th^ December 2012, a total of 53 inpatients met the case definition of *C. difficile*-associated infection.

**Figure 2:**
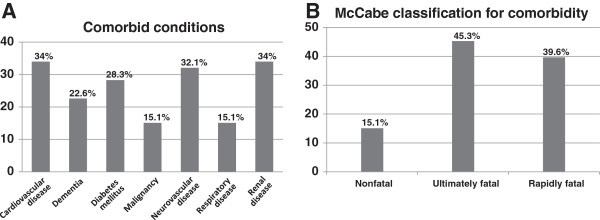
**Underlying comorbidity of the 53 patients with *Clostridium difficile*-associated infection during the outbreak period: A – Rate of each comorbid condition; B- Evaluation of the vital prognosis of the underlying clinical condition according to the McCabe score.**

Patient’s demographic characteristics are summarized in Table 1 and the co-morbid conditions are displayed in Figure 2A. Vital prognosis of the underlying clinical situation was evaluated according to the McCabe score, and overall only eight out of 53 (15.1%) patients had a nonfatal condition (Figure 2B).

**Table 1:** **Characteristics of the 53 patients with *Clostridium difficile*-associated diarrhea during the outbreak period**

**Patients (n = 53) characteristics**	**Number (%)**
Males	29 (54.7%)
Median age in years (interquartile range)	78.0 (71.0-86.0)
Place of origin	
Home	26 (49.1%)
Nursing home	11 (20.8%)
Long term care facility	16 (30.2%)
Hospital admission during the 6 previous months	35 (66.0%)
Antimicrobial exposure within 3-months before CDI diagnosis	47 (88.7%)
Gastric acid suppressors (Proton pump inhibitors) intake within 3-months before CDI diagnosis	49 (92.5%)
Clinical outcome	
Relapse	12 (22.6%)
Death at 6 months	34 (64.2%)
CDI Attributable cause	6 (11.3%)
CDI Contributing cause	19 (35.8%)
Unrelated	9 (17.0%)

CDI – *Clostridium difficile* infection.

Fifty patients (94.3%) had a positive toxin assay; of these, 6.0% (three patients) had at least one negative fecal toxin test in the seven days preceding the positive test, and 18.0% (nine patients) in the preceding 30 days. For these patients, the toxin test was repeated several times due to the persistent diarrhea. For the remaining three patients, CDI was diagnosed by means of detection of pseudomembranous colitis by fibrosigmoidoscopy and biopsy, since two of them had ileus, and the third one had a negative toxin test, but a high clinical suspicion [23]. The average time between patient’s admission and CDI diagnosis was 17 days, varying from one day to a maximum of 72 days.

**Figure 3:**
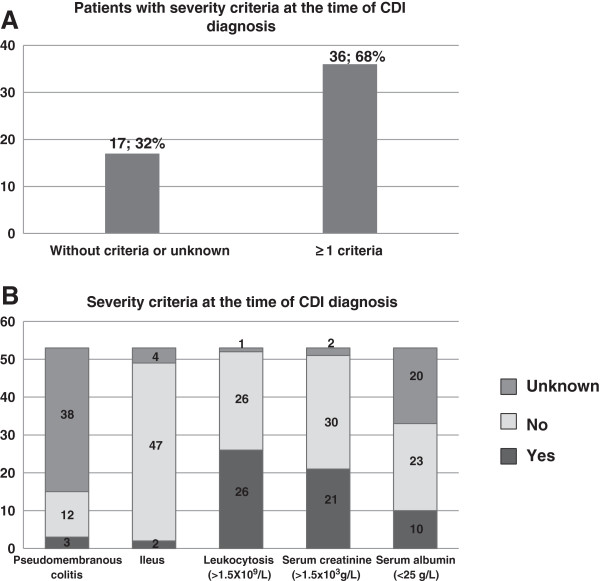
**Criteria for disease severity at the time of *Clostridium difficile *** **infection diagnosis: A – Rate of patients with no criteria or unknown criteria versus the rate of patients with at least one criteria of severe disease; B – Number of patients with, without or unknown status regarding the evaluated severity criteria: pseudomembranous colitis, ileus, leukocytosis, serum creatinine and serum albumin.**

Criteria for disease severity at the time of CDI diagnosis were based on the ESCMID guidance document; 68.0% had at least one criterion, where renal failure and leukocitosis were the most frequent (Figures 3A and 3B).

According to patient’s hospital medical records, 47 (88.7%) of the 53 cases had received antibiotics in the three months prior to the diagnosis, and among these, all except one had received two or more classes of antibiotics. The most commonly used antibiotics were fluoroquinolones (mainly ciprofloxacin) and penicillins in association with clavulanic acid or tazobactam (59.6% each), cephalosporins (36.2%), aminoglicosides (31.9%) and carbapenems (23.4%). In addition, in the same period of time, 49 (92.5%) patients had been prescribed with proton pump inhibitors.

Concerning the CDI onset, cases were classified according to standardized published definitions [12,13], and were distributed as follows: 30 (56.6%) cases were healthcare facility onset-healthcare facility associated (HCFA), 12 (22.6%) were community onset-HCFA, four (7.5%) cases were community onset, and seven (13.2%) cases had an indeterminate onset.

### Incidence rates and epidemic curve

**Figure 4:**
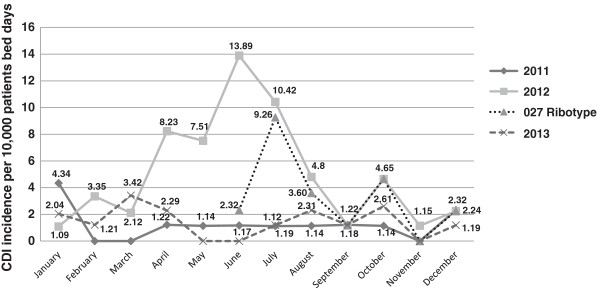
**Incidence of *Clostridium difficile *** **infection cases during the outbreak period (2012, from 1**^**st **^**January to 31**^**st **^**December), previous year (2011) and following year (2013).**

The CDI rate, calculated as the number of CDI cases per 10,000 patient bed days, increased from 1.09, in January 2012, to 8.23 in April 2012, when the outbreak was declared, with a peak of 13.89 in June 2012, with a decreasing trend afterwards. The epidemic curve of the outbreak, as well as the incidence curves in the previous (2011) and following (2013) years, are displayed in Figure 4. The end of the outbreak scenario was declared in 31^st^ January 2013, after three consecutive months in which the observed incidence rate was similar to the pre-outbreak situation.

### Treatment and clinical outcome

The global mortality within 30 days of the diagnosis of CDI was 39.6% (21/53) and further 13 patients died during the following 6 months, with a total crude mortality of 64.2% (34/53). A total of 24 patients (45.3%) died during the first CDI episode. From the 29 patients who survived the first episode, 12 relapsed (41.4%; 22.6% overall relapse rate) and 10 of these eventually died. After two independently clinical judgments, CDI was considered the attributable cause of death in six (17.6%; 11.3% overall attributable death) patients, a contributing cause in 19 (55.9%) patients and unrelated in nine (26.5%) cases.

The treatment of infected patients was established according to CHA protocol, which is based in the ESCMID guidelines [11]: 48 patients were treated either with metronidazole (n = 18) or vancomycin (n = 19), or with both vancomycin and metronidazole (n = 11); five patients did not receive treatment (four died before they could start any treatment, and one patient with mild disease was dismissed and CDI resolved without treatment).

Death rate during the first episode of CDI was higher in the group of patients treated with vancomycin only (9/19, 47.4%), followed by the patients treated with both antibiotics (5/11, 45.5%) and by those treated with metronidazole only (6/18, 33.3%).

**Figure 5:**
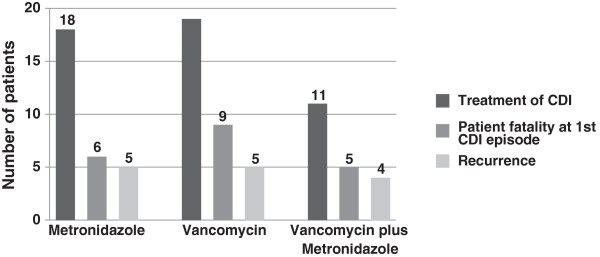
**Comparison of patient fatality during the first episode of *Clostridium difficile *** **infection and recurrence, according to the received treatment.**

The group with the highest recurrence rate was the one treated with both metronidazole and vancomycin (4/11; 36.4%), while recurrence rates were similar following treatment with either metronidazole (5/18; 27.8%) or vancomycin (5/19; 26.3%) (Figure 5).

In order to evaluate the CDI clinical burden, we compared the 25 cases for which CDI was the direct cause of (or contributed to) the death (group 1), with the cases for which no death occurred (group 2) (n = 19). The median age of patients in group 1 was significantly higher than that of group 2 (80.2 years; interquartile range 76 to 87 years *versus* 74.2 years; interquartile range 67 to 81 years; *P* = 0.022). Other traits that differentiated these two groups of patients concerned the severity of the underlying clinical situation, as well as the markers of severe CDI, such as renal failure and leukocytosis [24]. Indeed, taking into account the McCabe score, the rapidly fatal condition was observed in 56.0% (n = 14) of the patients of group 1, against 26.3% (n = 5) of the group 2 (*P* = 0.025). Also, both leukocytosis (19/25, 76% versus 6/19, 31.6%; *P* = 0.008) and renal failure (9/25; 36% versus 5/19; 26.3%; *P* > 0.05) were more frequent among group 1 than among group 2. As previously mentioned, in some cases, there was a considerable delay between patient’s admission and CDI diagnosis. Analyzing the two groups concerning this parameter, we observed that the average time for group 1 was 13.5 days (SD: 17.3) while for group 2 was 8.3 days (SD: 9.3), although this difference was not statistically significant.

### Strain typing

Among the 22 samples that were sent to the NIH reference laboratory for culture and typing, 20 had a positive faecal culture for *C. difficile*. Regarding the ribotype, 16 (72.7%) belonged to ribotype 027, and the remaining four strains belonged to one of each ribotype, AI-84, 056, 020 and 014. All the studied isolates belonging to ribotype 027 were detected from HCFA cases, while the isolates from ribotypes 014 and AI-84 corresponded to indeterminate onset cases.

All the isolates were positive for both the enterotoxin A and the cytotoxin B encoding genes. Binary toxin gene and the 18-bp deletion in the *tcdC* gene were present in the all 027 isolates and absent in the other ribotypes.

The *in vitro* resistance to moxifloxacin was confirmed for all 16 isolates of the 027 ribotype, (minimum inhibitory concentration (MIC) ≥32 mg/L), while the other non-027 ribotype isolates were susceptible to this antibiotic (MICs of 0.5 and 0.75 mg/L).

## Discussion

To date, little information is available concerning *C. difficile* infection in Portugal. The few existing studies were published in non-indexed national journals, and report data on hospital casuistic. Nevertheless, they showed an important increase on CDI cases, as well on mortality rates, over the last five years. None of these studies identified the infecting strain.

The *C. difficile* 027 ribotype strain implicated in this outbreak showed traits that are common to the so called hypervirulent strains of this ribotype, including the known mutation in the *tcdC* gene and the presence of the binary toxin. Also relevant is the reduced susceptibility to fluoroquinolones of these strains, which likely contributes to their rapid dissemination [25] compared to non-outbreak isolates of the same type. Indeed, several studies point to fluoroquinolones therapy as a risk factor for *C. difficile*-associated disease during outbreaks [16,26-28]. In the present cohort, 60% of the patients had a history of recent fluoroquinolones consumption, which has likely contributed to the spread of the epidemic strain, once it was introduced in the hospital.

The antibiotic stewardship implemented after the outbreak was declared, with special focus on reduced prescriptions of fluoroquinolones and third generation cephalosporins (Figure 1), presumably contributed to the progressive reduction in the incidence of CDI (Figure 4). This fact highlights the value of this measure to control nosocomial CDI, in agreement with previously published studies [16,29,30].

It was not possible to trace how this epidemic ribotype 027 *C. difficile* strain was introduced in the hospital, as the first samples were not sent to the NIH for ribotyping, hampering the identification of the index case. Moreover we were not able to associate the presence of this strain with the occurrence of other CDI cases in Portugal (outbreak or sporadic cases) probably due to the absence of a functional notification system of this disease. Nevertheless, it is worthwhile to underline the implication of *C. difficile* 027 strain in the present outbreak, in contrast to the declining trend reported across Europe concerning the prevalence of this strain in favor of another emergent ribotypes [10].

To date there is no consensus regarding the association of *C. difficile* ribotype 027 strains with more serious clinical outcome. Indeed, while most of the hospitals with 027 strain outbreaks have reported increased severity of the associated-disease, others are showing no difference to other ribotypes [16,31,32].

In the outbreak here described, 68% of the patients had at least one criterion of severe disease at diagnosis. Also, mortality occurred at high rate, with a 30-day all-cause mortality of 39.6% and a 027-CDI-related mortality rate of 18%, which is considerable higher than that reported in other 027-associated outbreaks [16,33-36]. Nevertheless, our data suggests that, besides the infecting strain and its ability for rapid spreading when antibiotic pressure is present, other factors have contributed to this dramatic mortality rate such as advanced age, co-morbidities and the co-existence of rapidly fatal conditions.

Following antibiotic treatment for CDI, the highest mortality rate occurred for the vancomycin-treated patients, followed by the patients treated with vancomycin plus metronidazole, and the highest recurrence rate was also observed for these patients, which was an unexpected finding [37]. The rationale for observing higher rates of treatment failure with vancomycin is not clear, but can be probably attributed to the fact that those patients had already a very poor prognostic, as also pointed by Gravel *et al.*[38]. Also, the worse outcome for patients treated with vancomycin or both vancomycin and metronidazole may be due to the clinical severity of CDI which led to the choice of the treatment according to published guidelines [23].

It is worth mentioning that we did not evaluate which patients had received non-CDI antimicrobials during and/or after *C. difficile* treatment, as well as differences in antibiotic regimens. This likely implicated the disease outcome, as previous highlighted [39].

Concerning the CDI onset classification, we point out that patients classified as being CDI community onset, all came from nursing homes or other long term care facilities, so all CDI cases should be considered healthcare associated.

Aiming to control the outbreak, the ICT conducted an investigation that identified several problems concerning CDI patients, namely: previous and continuous excess use of broad spectrum antibiotics and proton pump inhibitors, with no clear indication registered in the clinical process; low level of clinical suspicion; and low sensibility of the diagnostic test used, contributing to late diagnosis of CDI, and consequently to a delay in the establishment of therapy and to a late implementation of control measures. In this context, it is worth emphasizing that the laboratory diagnosis of CDI was performed with a stand-alone rapid enzyme immunoassay for the *C. difficile* toxins, which has originated a number of false negative results, hampering the management of the CDI [40]. In addition, at the beginning of the outbreak investigation, the OCT detected that in some cases clinicians did not registered severity criteria of CDI. Notably the presence of renal failure and leukocytosis were not always adequately valorized. A recent study showed that both these clinical conditions are predictors of severe CDI if measured on the day of diagnosis [24]. To correct these findings, a protocol for the initial evaluation and treatment of all patients with diarrhea was implemented since May 2012.

It should also be pointed out the lack of awareness and knowledge of the institutional guidelines by the hospital staff, especially among clinicians, indicating the need of a different approach to motivate HCW in infection control educational activities, which are not mandatory in Portugal. The bundle of measures that were taken tried to face and correct the detected problems, together with the involvement of all HCW in their resolution.

We therefore considered that all these constraints, together with the presence of a hypervirulent strain with a high spread capacity, contributed to the increased risk of nosocomial transmission and to the severity of CDI episodes observed in this outbreak.

## Conclusion

In conclusion, regarding the infecting strain, while the high potential for spreading of 027 strains seems clear, further investigation on epidemic strains can be a useful contribution to shed some light on the virulence and epidemic potential of *C. difficile* 027, in accordance with recent studies suggesting that subtypes may influence the severity of clinical outcome.

High level suspicion and early diagnosis of CDI, with initial correct evaluation of severity criteria, and adequate treatment according to this evaluation, together with the reinforcement of a bundle of infection control measures is necessary to avoid the spread of *C. difficile.* Antibiotic stewardship is perhaps the most important isolated measure that can be taken to prevent CDI related clinical burden, but it is difficult to accomplish in a growing population of elderly, dependent and institutionalized patients with consecutive healthcare associated infections.

Finally, this is the first outbreak of *C. difficile* due to ribotype 027 strain that is reported in Portugal, highlighting the importance of implementing a close surveillance of CDI in Portugal, both at population-based and nosocomial levels.

## Competing interests

The authors declare no competing of interest.

## Authors' contributions

MO conceived and carried out experiments and analysed data; MC analysed data; MG and SC carried out experiments and analysed data; SM analysed data; AS and JR carried out experiments; DF conceived and analysed data. All authors were involved in writing the paper and gave final approval to the submitted version.

## Pre-publication history

The pre-publication history for this paper can be accessed here:

http://www.biomedcentral.com/1471-2334/14/209/prepub
